# Cryptotanshinone suppresses cell proliferation and glucose metabolism via STAT3/SIRT3 signaling pathway in ovarian cancer cells

**DOI:** 10.1002/cam4.1691

**Published:** 2018-08-09

**Authors:** Yufei Yang, Yue Cao, Lihua Chen, Fei Liu, Zihao Qi, Xi Cheng, Ziliang Wang

**Affiliations:** ^1^ Cancer Institute Fudan University Shanghai Cancer Center Shanghai China; ^2^ Department of Gynecological Oncology Fudan University Shanghai Cancer Center Shanghai China; ^3^ Department of Oncology Shanghai Medical College Fudan University Shanghai China

**Keywords:** cryptotanshinone, glucose metabolism, ovarian cancer, SIRT3

## Abstract

Ovarian cancer is the most malignant gynecologic cancer among women worldwide. Cryptotanshinone (CT), isolated from Salvia miltiorrhiza Bunge, has been identified as a potential therapeutic agent in treating several malignant tumors, but the molecular mechanism of CT in ovarian cancer still remains illustrated. Here, we sought to elucidate the regulatory function of CT on cell glucose metabolism in ovarian cancer. The treatment of CT on ovarian cancer cells effectively inhibited glucose uptake and lactate production in ovarian cancer cells. The expression levels of glycolysis‐related proteins, such as GLUT1, LDHA, and HK2, were decreased by the treatment of CT detected by qRT‐PCR and immunoblotting. Mechanistically, CT exerted its anti‐tumor effect by targeting STAT3/SIRT3/HIF‐1α signaling pathway in vitro and in vivo, which could be rescued by the introduction of SIRT3 shRNA in ovarian cancer cells. The clinical data showed that the expression level of STAT3 in ovarian cancer patients’ sera and tissues was positively correlated with those of GLUT1, LDHA, HK2 and HIF‐1α, but negatively with that of SIRT3These findings provide evidence that CT inhibited cellular glycolysis‐induced cell growth and proliferation through repression of STAT3/SIRT3/HIF‐1α signaling pathway, indicating that CT may be developed as a chemotherapeutic agent to treat ovarian cancer.

## Abstract 2

Cryptotanshinone (CT) effectively inhibited glucose uptake and lactate production in ovarian cancer cells. CT exerted its anti‐tumor effect by targeting STAT3/SIRT3/HIF‐1α signaling pathway in vitro and in vivo. CT may be developed as a chemotherapeutic agent to treat ovarian cancer.

## 1 INTRODUCTION

Epithelial ovarian cancer (EOC) was the most prevalent gynecologic malignancies worldwide.^1^, ^2^ As ovarian tumors at the early stage have no specific symptoms, most of EOC patients are diagnosed at advanced phase of disease, and the overall five‐year survival rate is relatively low.^3^, ^4^ At current, the standard first‐line treatment for EOC is primarily cytoreductive surgery (CRS), which was followed by platinum‐based chemotherapy.^5^ For most advanced EOC patients, the production of drug resistance was one of the main causes of treatment failure.^6^ Therefore, it is imperative to find useful therapeutic agents to improve the survival rate of EOC patients and to decrease the failure of chemotherapy.

Cryptotanshinone (CT) is isolated from the plant Salvia miltiorrhiza Bunge and has been identified to block STAT3 phosphorylation and homodimerization.^7^, ^8^, ^9^ CT has been demonstrated to have anti‐tumor activities in various malignancies, including breast cancer,liver cancer,prostate cancer, and ovarian cancer.^10^, ^11^, ^12^, ^13^ CT was found to induced apoptosis and cell cycle arrest in gastric cancer cells via reactive oxygen species (ROS)‐mediated MAPK and AKT signaling pathways.^14^, ^15^ It has been reported that CT was a potent STAT3 inhibitor and inhibited STAT3 Tyr705 phosphorylation effectively in DU145 prostate cancer cells.^16^ Therefore, growing evidence indicates that cryptotanshinone is a potent anticancer agent targeting STAT3 protein.

STAT3 (signal transducer and activator of transcription 3) promotes multiple signaling pathways involving the mediation of cell proliferation, invasion, epithelial‐mesenchymal transition (EMT) and angiogenesis and has been considered as a key target for cancer therapy.^17^, ^18^, ^19^ Increasing evidence has shown the existence of genetic variation and amplification of STAT3 in major malignancies.^19^, ^20^, ^21^, ^22^ STAT3 signaling pathway plays a vital role in cancer progression and tumor microenvironment,^23^, ^24^ indicating that it may be used as a therapeutic target.

SIRT3 is one of the sirtuins of NAD‐dependent deacetylases and located in mitochondria.^25^ SIRT3 is involved in the processes of energy metabolism and development of diseases including the cancer, cardiovascular, and nervous systems.^26^, ^27^, ^28^ SIRT3 adjusts several mitochondrial functions,^29^, ^30^ such as managing ROS, ATP production, and cell death. In the previous studies, it has been reported that the expression level of SIRT3 suppressed in several malignant tumor,^31^, ^32^, ^33^ including prostate, lung, and gastric cancers.

To evaluate the function and mechanism of CT on glucose metabolism in ovarian cancer cells, we tested the inhibitory ratio of CT on ovarian cancer cells and the effect of CT on glucose uptake and lactate production in ovarian cancer cells. We found that CT inhibited glucose metabolism in ovarian cancer cells through inducing SIRT3/HIF‐1α signaling pathway.

## 2 MATERIAL AND METHODS

### 2.1 Cell lines and cell culture

Human ovarian cancer cell lines, Hey and A2780, were obtained from the Cell Bank of the Chinese Academy of Science. The cells were grown in Dulbecco's modified Eagle's medium (DMEM, HyClone, Thermo Scientific, Waltham, MA) supplemented with 10% fetal bovine serum (Gibco, Life technologies, Carlsbad, CA), 100 U/mL penicillin (Biowest, Nuaillé, France), and 100 U/mL streptomycin (Biowest, Nuaillé, France) and incubated at 37°C in a humidified atmosphere with 5% CO_2_.

### 2.2 Drug treatment

CT (Catalog No. S2285, Selleck, USA) were dissolved in DMSO and kept in ﹣20°. Paclitaxel was purchased from BristolMyers Squibb Company (NY, USA). All ovarian cells were treated with different concentrations (nmol/L) CT for 48 hours.

### 2.3 Plasmid construction and cell transfection

The DNA oligonucleotides designed to generate short hairpin RNAs against the open reading frame of SIRT3 mRNA were 5′‐AAGTGGAGGCAGCAGTGACAA‐3′(shRNA1) and 5′‐CTTGAGAGAGTGTCGGGCATC‐3′ (shRNA2). The sequences of STAT3 shRNA3 were 5′‐ TAGAGAATCTCCAGGATGACT‐3′ (shRNA1) and 5′‐CAACAGATTGCCTGCATTGGA‐3′ (shRNA2).

The recombinant plasmids, pLKO/shSIRT3‐1, pLKO/shSIRT3‐2, pLKO/shSTAT3‐1, and pLKO/shSTAT3‐2, were generated according to the previously reported method.^34^ The control vector was similarly constructed by directly inserting oligonucleotides encoding short hairpin RNA against green fluorescence protein mRNA(shGFP) into pLKO.1 vector.

Lentivirus carrying SIRT3 shRNA or STAT3 was generated and harvested as described previously.^34^ Briefly, the cells were infected twice for a total of 4 days (2 days for each infection), and the positive clones were selected with puro23in (200 ng/mL) for 7‐10 days. Control cell lines were generated by infection with viruses containing the empty vector following the same protocol.

Recombinant plasmids, containing human full length of cDNA sequences of pENTER‐STAT3, were purchased from Vigene Biosciences (Jinan, China). STAT3 cDNA was subcloned into pcDNA3.1 (−) expression vector (Invitrogen, Carlsbad, CA, USA), generating the recombinant plasmids pcDNA3.1 (−)‐STAT3. The empty pcDNA3.1 (−) was used as a control. Hey and A2780 cells were transfected withpcDNA3.1 (−)‐STAT3 or control empty pcDNA3.1 (−) using Lipofectamine 2000 according to the manufacturer's instructions.

### 2.4 Glycolysis analysis

Glucose and lactate levels in culture media were measured using the BioProfile FLEX analyzer (Nova Biomedical, Waltham, MA) and normalized to cell number or using the Lactate Reagent Kit (Trinity Biosciences, Co Wicklow, Ireland). Fresh media were added to a 6‐well plate of subconfluent cells, and lactate production in the media was measured 30‐60 minutes (Lactate Reagent Kit) or 6‐24 hours (BioProfile Analyzer) later and normalized to the number of cells in each well.

### 2.5 Western blot analysis

Western blot analysis was performed to determine the expression levels of various proteins in cells. Cells were harvested, washed with cold 1× PBS, and lysed with RIPA lysis buffer (Beyotime) for 30 minutes and then centrifuged at 12 000 *g* for 15 minutes at 4°C. The total protein concentration was determined by BCA protein assay kit (Beyotime, P 0011). Equal amounts (30 μg per load) of protein samples were subjected to SDS‐PAGE electrophoresis and transferred on to polyvinylidene fluoride (PVDF) membranes (Millipore, IPVH00010). The blots were blocked in 10% nonfat milk, and incubated with primary antibodies, followed by incubation with secondary antibodies conjugated with horseradish peroxidase (HRP). The protein bands were developed with the chemiluminescent reagents (Millipore). Antibodies to STAT3 (10253‐2‐AP), GLUTl (21829‐1‐AP), LDHA (19987‐1‐AP), HK2 (22029‐1‐AP), SIRT3 (10099‐1‐AP), and HIF‐1α (20960‐1‐AP) were purchased from Proteintech^™^. The antibody to β‐actin (A5441) was obtained from Sigma‐Aldrich.

### 2.6 Luciferase reporter assay

Human promoter sequences of sirt3 were inserted into a pGL3‐basic vector as pGL3‐ S3‐Promoter. One hundred nanograms of constructed plasmid and 5 ng renilla luciferase control plasmid were transfected into cells expressing Hey/shSIRT3‐1 and A2780/shSIRT3‐1 in 6‐well plates. Dual Luciferase assay kit (Promega, Madison, WI, USA) was used for the detection of luciferase activities at 48 hours after transfection. Reporter luciferase activities were normalized based on Renilla luciferase and then rescaled to vector control signals equal to unit 1. All experiments were repeated at least three times. Data represent mean fold change (± SD; n = 3) relative to the control.

### 2.7 Tissue and serum samples

Ethical approval for the study was obtained from the Clinical Research Ethics Committee of Fudan University Shanghai Cancer Center (FUSCC). Fresh tissues from 38 ovarian cancer patients who had undergone surgery at FUSCC between June 2016 and January 2017 and 22 age‐ and sex‐matched healthy individuals enrolled as a control group were included for RNA preparation. We also collected 45 blood samples in patients with ovarian cancer before surgery. Meanwhile, 33 age‐ and sex‐matched healthy individuals were enrolled as a control group. These healthy individuals underwent medical examination to exclude the evidence of tumor and other metabolism‐associated diseases. All sera were collected using standard procedures.

### 2.8 In vivo tumor growth assay

Animal experiments were approved by the Ethics Committee at FUSCC. Briefly, female BALB/c nude (Shanghai Slac Laboratory Animal Co. Ltd, 4‐6 weeks) were injected subcutaneously with Hey cells (5 × 10^6^ suspended in 0.1 mL PBS for each mouse). Once reaching an average tumor volume of 100 mm^3^, mice were randomized into groups (n = 5). Before treated with CT, all the mice were subjected to perform positron emission tomography/computed tomography (PET/CT) scan. The glucose uptake of tumor was evaluated by the standard uptake value (SUV). Then they were intraperitoneally treated with CT (10 mg/kg) thereafter. Administration of vehicle or agents and measurement of tumor growth with a digital caliper were performed once every other day. Tumor volumes were calculated with the following equation: *V* = *L* × *W* ^2 ^× 0.52. *V* represented the volume, *L* represented length, and *W* was the width. Mice were sacrificed, and the tumors were dissected and weighed 1 week after the last CT injection. RT‐PCR of xenograft tumor was performed according to the protocol above.

### 2.9 Statistical analysis

Statistical analysis was performed using SPSS software (version 18.0). Student's *t* test or ANOVA was used to compare quantitative data, and chi‐square test or Kruskal‐Wallis Tests were used to analyze enumeration data. Progression‐free survival and overall survival curves were plotted using the Kaplan‐Meier method and were analyzed by the log‐rank test. *P *<* *0.05 (two tailed) was considered statistically significant.

## 3 RESULTS

### 3.1 CT inhibits cell proliferation and glycolysis in ovarian cancer

**Figure 1:**
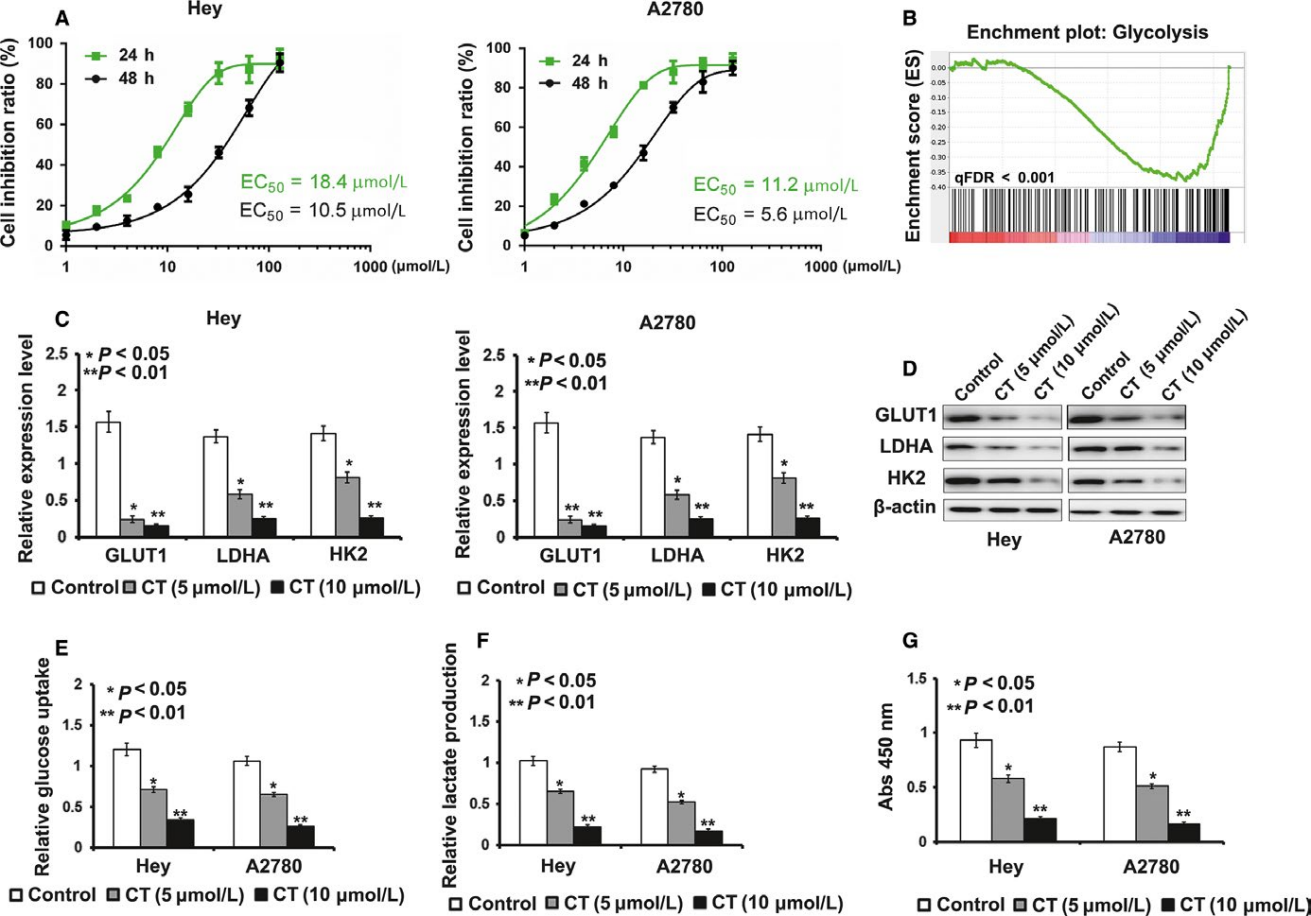
Cryptotanshinone (CT) suppresses cell proliferation by inhibiting cell glycolysis. A, In Hey and A2780cell lines, exposure to bufalin resulted in significant decrease in cell viability compared with control in a dose‐dependent manner. B, Analysis of cell function regulated by gene‐chip assay. C‐D, The results of real‐time PCR and Western blot exhibited that mRNA level and protein level of GLUT1, LDHA, and HK2 were down‐regulated by treatment of CT (*P <* *0.05, **P < 0.01). E, The treatment of CT caused glucose uptake decreased in Hey and A2780 cells when compared to controls (*P <* *0.05, **P < 0.01). F, Lactate production was reduced by the treatment of CT in Hey and A2780 cells when compared to controls (*P < 0.05, **P < 0.01). G, The results of Colony formation assay exhibited that CT's inhibitory effect on colony formation ability was obviously weakened by the treatment of CT when compared to controls (*P < 0.05, **P < 0.01)

Ovarian cancer cells, Hey and A2780, were treated with a series of concentrations of CT (shown in Figure 1A) for 24 and 48 hours, respectively. In both cancer cell lines, exposure to cryptotanshinone significantly decreased cell viability compared with controls in a dose‐dependent and time‐dependent manner (*P* < 0.05, Figure 1A). The half‐maximal inhibitory concentration (IC_50_) value for Hey and A2780 cells was 18.4 (95%CI 17.59‐23.65) μM and 11.2 (95%CI 10.2‐24.96) μM, respectively. Concentrations used in subsequent experiments were calculated from above dose‐response curves.

To explore the effect of CT on the pathogenesis of ovarian cancer, we used gene‐chip assays to compare the expression of CT‐induced genes in Hey cells treated by CT and control cells. Through this approach, we found that genes involved in glycolysis were enriched in cells treated with CT, compared with the corresponding controls (Figure 1B). Furthermore, we used qRT‐PCR and Western blotting to detect the expression levels of glycolysis‐related proteins in Hey and A2780 cells after CT treatment to validate the results of gene‐chip assay. Compared with their controls, the results of qRT‐PCR and Western blotting exhibited that mRNA and protein expression levels of GLUT1, LDHA, and HK2 in Hey and A2780 cells treated by CT were decreased (*P* < 0.05) (Figure 1C,D). Subsequently, we performed MTT, glucose uptake, and lactate production assays to detect the effect of CT on cell proliferation and glycolysis in ovarian cancer cells. As shown in Figure 1E,F, CT decreased glucose uptake and lactate production levels in Hey and A2780 cells in a dose‐dependent manner, compared with their control cells.

### 3.2 CT regulates glucose metabolism by inducing the expression of SIRT3 in ovarian cancer cells

**Figure 2:**
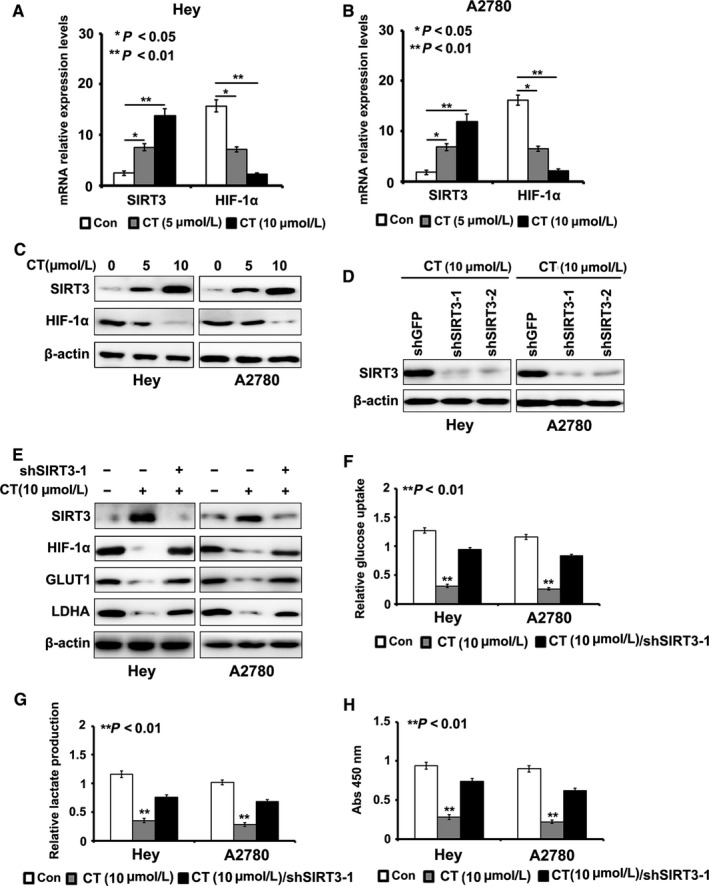
Cryptotanshinone (CT) inhibits cell glycolysis by enhancing the expression of SIRT3. A‐C, The mRNA level and protein level of SIRT3 and HIF‐1α was suppressed after CT treatment in a dose‐dependent manner. D, Silencing effect of SIRT3 with shRNAs was detected by Western blot. E, Knockdown of SIRT3 rescued the effect of CT on the expression of SIRT3, HIF‐1α, GLUT1, and LDHA detected by Western blot. F, Silencing of SIRT3 rescued the effect of CT on cells in glucose uptake assays when compared to their controls treated with bufalin (*P *<* *0.01). G, Silencing of SIRT3 rescued the effect of CT on cells in lactate production assays when compared to their controls treated with bufalin (*P *<* *0.01). H, The results of colony formation assay exhibited that CT's inhibitory effect on colony formation ability was obviously enhanced by the induction of SIRT3 shRNA when compared to controls (*P *<* *0.01)

It has been reported that SIRT3 played vital roles in cell glycolysis by regulating the expression of HIF‐1α.^35^ We found that the mRNA and protein level of SIRT3 and HIF‐1α were decreased after CT treatment in a dose‐dependent manner, demonstrating SIRT3 might be a downstream effector of STAT3 in Hey and A2780 cells (Figure 2A‐C) (*P *<* *0.05). To further confirm the role of SIRT3 in CT‐induced glycolysis inhibition, we established Hey/shSIRT3 and A2780/shSIRT3 cells. As shown in Figure 2D, the effect of silencing of SIRT3 shRNA‐1 was better than that of SIRT3 shRNA‐2, so we took SIRT3 shRNA‐1 to perform subsequent experiments.

Next, we introduced SIRT3 shRNA‐1 into Hey and A2780 cells, which were treated with CT (10μΜ). We found that silencing of SIRT3 effectively rescued the effect of CT on the expression of HIF‐1α, GLUT1, and LDHA (Figure 2E). We also found that SIRT3 knockdown effectively rescued the inhibitory effect of CT on glucose uptake and lactate production in ovarian cancer cells when compared to their controls (*P *<* *0.05, Figure 2F,G). The results of MTT assay showed that CT's inhibitory effect on ovarian cancer cell proliferation was obviously enhanced by the induction of SIRT3 shRNA‐1 when compared to controls (*P *<* *0.05, Figure 2H).

### 3.3 CT inhibits the promoter activity of SIRT3 by suppressing STAT3 in ovarian cancer cells

**Figure 3:**
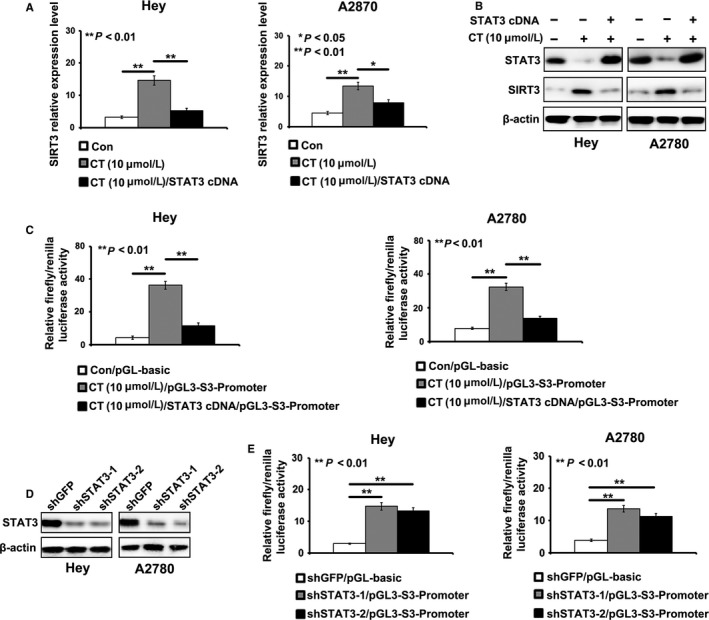
Cryptotanshinone (CT) suppresses the promoter activity of SITR3 by inhibiting STAT3. A‐B, STAT3 overexpression rescued the induction of CT on the expression of SIRT3 detected by real‐time PCR and Western blot (*P <* *0.05, **P < 0.01). C, STAT3 overexpression reduced the enhancing effect of CT on the promoter activity of SIRT3 detected by dual‐luciferase reporter gene assay in Hey and A2780 cells (**P < 0.01). D, Silencing effect of STAT3 with shRNAs was detected by Western blot. E, Knockdown of STAT3 reduced the promoter activity of SIRT3 in Hey and A2780 cells (**P < 0.01)

As STAT3 was a potential target for CT, we inferred that CT might regulate the expression of SIRT3 by suppressing STAT3. To verify our hypothesis, we introduced STAT3 cDNA into Hey and A2780 cells treated by CT (10 μΜ). We found that overexpression of STAT3 rescued the inhibitory effect of CT on the expression of SIRT3 detected by qRT‐PCR and Western blotting (Figure 3A,B).

To further illustrate the regulatory mechanism of CT on SIRT3, we cloned the promoter sequences of SIRT3 into pGL3‐basic vector and reconstructed the recombinant plasmid, pGL3‐S3‐promoter. We introduced pGL3‐S3‐promoter into Hey and A2780 cells treated by CT (10 μΜ). We found that the treatment of CT obviously reduced luciferase activities induced by SIRT3 promoter in Hey and A2780 cells, which was recused by the introduction of STAT3 cDNA (Figure 3C). Silencing of STAT3 also decreased the luciferase activities induced by SIRT3 promoter in Hey and A2780 cells (Figure 3D,E). These results showed that CT inhibited cell glycolysis through suppressed STAT3/SIRT3 pathway in ovarian cancer cells.

### 3.4 CT inhibits cell proliferation and glycolysis of ovarian cancer cells in vivo 

**Figure 4:**
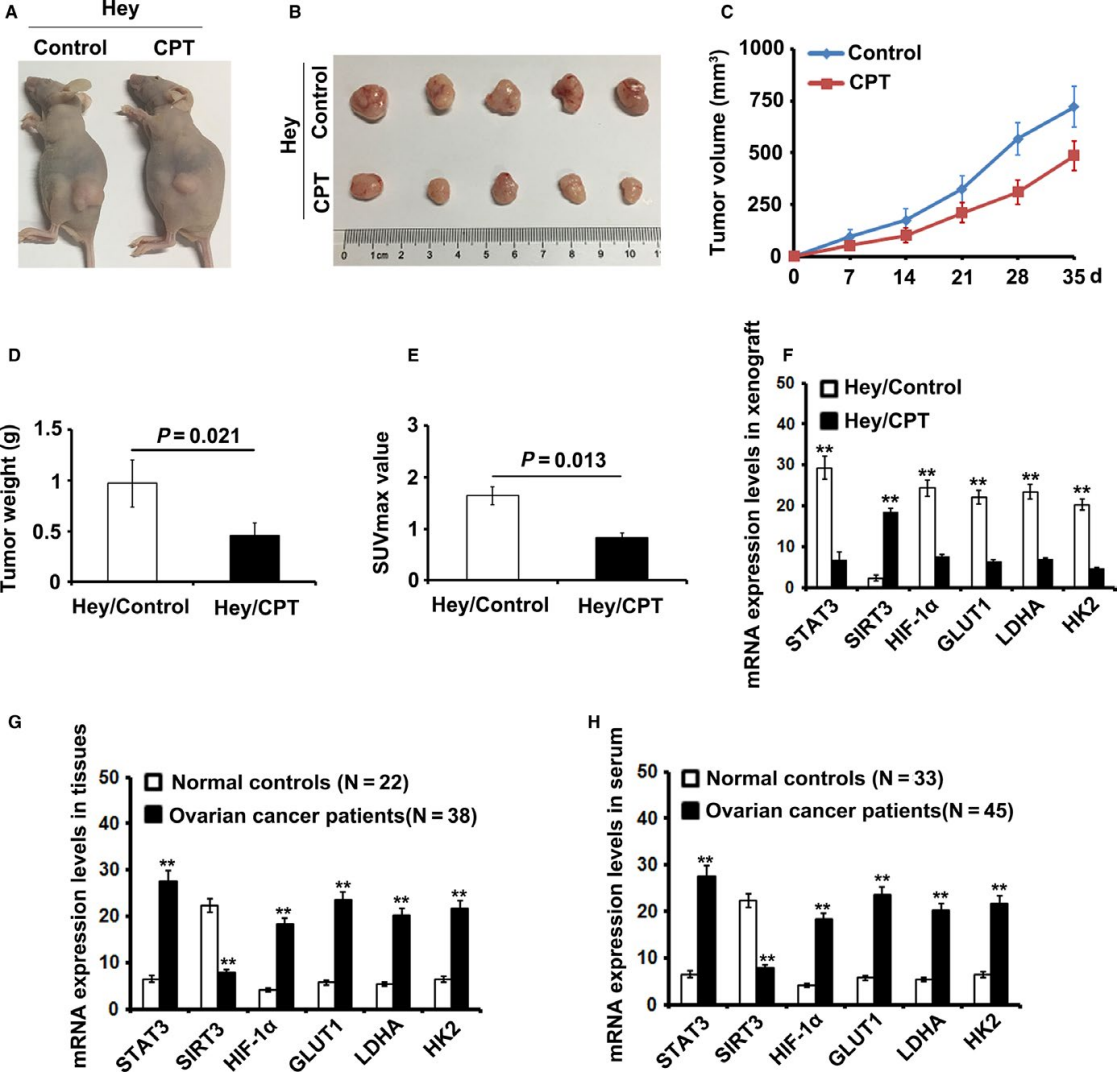
Cryptotanshinone (CT) inhibits cell proliferation and glycolysis of ovarian cancer cells by inducing SIRT3/HIF‐1α signaling pathway in vivo. A‐B, Treatment with CT slowed the speed of tumor growth in vivo (*P* < 0.05). C‐D, The tumor volume and weight in the experimental group were significantly lower than those in control group (*P* < 0.05). E, CT can significantly suppressed the glucose uptake in ovarian cancer in vivo and resulted in a lower SUV (*P* < 0.05). F, The expression levels of STAT3, SIRT3, HIF‐1α, GLUT1, LDHA, and HK2 was detected by real‐time PCR in xenograft tumors and the corresponding controls (**P < 0.01). G, The expression levels of STAT3, SIRT3, HIF‐1α, GLUT1, LDHA, and HK2 were detected by real‐time PCR in ovarian cancer patients’ tissues and normal tissues (**P < 0.01). H, The expression levels of STAT3, SIRT3, HIF‐1α, GLUT1, LDHA, and HK2 was detected by real‐time PCR in ovarian cancer patients’ serum and normal controls (**P < 0.01)

To test the antitumor effect of CT in vivo, we injected Hey cells into nude mice to observe subcutaneous tumor formation. After the volume of the tumor reached 100 mm^3^, the mice were subjected to CT treatment every other day. As shown in Figure 4A,B, the treatment with CT obviously slowed the growth speed of xenografts in vivo*,* compared with their controls. The tumor volume and weight in the mouse treated by CT were significantly lower than those in control group (*P* < 0.05, Figure 4C,D). In addition, based on the detection results of PET‐CT, we found that CT significantly suppressed the glucose uptake in ovarian cancer in vivo and resulted in a lower SUV max value (Figure 4E,F). To determine whether the CT inhibited the expression of STAT3, SIRT3, HIF‐1α, GLUT1, LDHA, and HK2 in vivo, we performed qRT‐PCR to detect these genes expression in tumor tissues from the mouse with or without the treatment of CT. As shown in Figure 4G, the expression levels of STAT3, HIF‐1α, GLUT1, LDHA, and HK2 were largely reduced, but the expression of SIRT3 was enhanced, compared with the their controls.

### 3.5 The expression of STAT3, SITR3, HIF‐1α, GLUT1, LDHA, and HK2 in ovarian cancer patient tissues and serum

We tested the expression levels of STAT3, SITR3, HIF‐1α, GLUT1, LDHA, and HK2 in 38 ovarian cancer patient tissues and 22 normal tissues. The expression levels of SIRT3 were lower in the cancer patient tissues but higher in normal tissues, while the expression levels of STAT3, HIF‐1α, GLUT1, LDHA, and HK2 were higher in tumor tissues but lower in normal tissues. We also compared the expression levels of STAT3, SITR3, HIF‐1α, GLUT1, LDHA, and HK2 of 44 ovarian cancer patient serums with 35 normal controls. We found that the expression level of SIRT3 was negative with those of STAT3, HIF‐1α, GLUT1, LDHA, and HK2 in patient serums, compared with the corresponding controls.

## 4 DISCUSSION

In this study, we described the inhibitory effects of CT on cell proliferation and glycolysis in ovarian cancer cells through suppressing STAT3/SIRT3 signaling pathway, which indicates that CT may be developed to be a potential drug for the treatment and prevention of ovarian cancer.

Despite the significant developments in detection and therapy of ovarian cancer during the past decades, ovarian cancer still remains a high rate of mortality. The most common chemotherapy was based on paclitaxel and platinum, but the patients still have a challenging problem of drug resistance.^36^ CT is one of the tanshinones isolated from Salvia miltiorrhiza Bunge, and now is widely used in several diseases, including high blood pressure, fibrosis, and several malignant tumors. CT was found to induce apoptosis and cell cycle arrest in gastric cancer (GC) cells via reactive oxygen species (ROS)‐mediated MAPK and AKT signaling pathways, and this CT may be a useful compound for the developing anticancer agents for GC.^14^ CT also suppressed the proliferation of prostate cancer cells via the inhibition of JNK/STAT3 signaling pathway.^16^ Meanwhile, CT was found to inhibit the proliferation of ovarian cancer cells and sensitize ovarian cancer cells to chemotherapy.^13^ In the present study, we demonstrated that CT had the anti‐cancer effects on ovarian cancer cells in a dose‐dependent manner. We further found that CT could reduce cell glycolysis level in ovarian cancer cells by inhibiting the glucose uptake and lactate production, which may further suppressed cancer cell growth and proliferation.

Previous studies disclosed that mTOR‐STAT3‐HK2 pathway is involved in the glycolysis of hepatocellular carcinoma (HCC) cells and STAT3 may regulate HCC glycolysis through HK2 pathway.^37^ Moreover, our results showed that STAT3 might directly suppress the promoter activity of SIRT3. SIRT3 is a key NAD+‐dependent protein deacetylase in the mitochondria of mammalian cells, functioning to prevent cell aging and transformation via regulation of mitochondrial metabolic homeostasis.^32^, ^33^ It has been reported that SIRT3 overexpression represses glycolysis and proliferation in breast cancer cells, providing a metabolic mechanism for tumor suppression.^38^ SIRT3 mediates metabolic reprogramming by destabilizing hypoxia‐inducible factor‐1α (HIF1α), a transcription factor that controls glycolytic gene expression.^35^ Consistent with previous studies, our results revealed the new evidence that SIRT3 may be a downstream target for STAT3 and STAT3 could regulate the expression of SIRT3 at the transcript level, which further affected the expression of glycolysis‐related proteins, such as GLUT1, LDHA, and HK2. Growing evidence showed that the curative effects of CTS on various cancers are accomplished mainly through modulating STAT3, so our results supplied new data to clarify the underlying mechanism of CTS on various cancers.

In conclusion, we demonstrated that CT inhibited cellular glycolysis‐induced cell growth and proliferation via suppressing STAT3/SIRT3/HIF‐1α signaling pathway. Our results not only provide the new insight into the underlying mechanism of CT‐induced glycolysis, but also offer the important implications for the development of therapeutic approaches using CT to prevent the recurrence in various cancers, including ovarian cancer.

## CONFLICT OF INTEREST

No potential conflicts of interests were disclosed.

## References

[1] Peres LC , Cushing‐Haugen KL , Kobel M , et al. Invasive epithelial ovarian cancer survival by histotype and disease stage. J Natl Cancer Inst. 2018 10.1093/jnci/djy071 PMC6335112 29718305

[2] Long J , Zhu JY , Liu YB , et al. Helicase POLQ‐like (HELQ) as a novel indicator of platinum‐based chemoresistance for epithelial ovarian cancer. Gynecol Oncol. 2018;149:341‐349. 29572031 10.1016/j.ygyno.2018.03.006

[3] Cole AL , Barber EL , Gogate A , Tran AQ , Wheeler SB . Economic analysis of neoadjuvant chemotherapy versus primary debulking surgery for advanced epithelial ovarian cancer using an aggressive surgical paradigm. Int J Gynecol Cancer. 2018;28(6):1077‐1084. 29683880 10.1097/IGC.0000000000001271 PMC6033676

[4] Dilley S , Erickson BK , Phillips CE , et al. Do differences in medical comorbidities and treatment impact racial disparities in epithelial ovarian cancer? Gynecol Oncol. 2018;149:49‐52. 29605050 10.1016/j.ygyno.2017.10.035 PMC7307692

[5] Shimokawa M , Kogawa T , Shimada T , et al. Overall survival and post‐progression survival are potent endpoint in phase III trials of second/third‐line chemotherapy for advanced or recurrent epithelial ovarian cancer. J Cancer. 2018;9:872‐879. 29581765 10.7150/jca.17664 PMC5868151

[6] Lim MC , Yoo HJ , Song YJ , et al. Survival outcomes after extensive cytoreductive surgery and selective neoadjuvant chemotherapy according to institutional criteria in bulky stage IIIC and IV epithelial ovarian cancer. J Gynecol Oncol. 2017;28:e48. 28541636 10.3802/jgo.2017.28.e48 PMC5447147

[7] Tang L , He S , Wang X , et al. Cryptotanshinone reduces psoriatic epidermal hyperplasia via inhibiting the activation of STAT3. Exp Dermatol. 2018;27:268‐275. 29427477 10.1111/exd.13511

[8] Wang Y , Lu HL , Liu YD , et al. Cryptotanshinone sensitizes antitumor effect of paclitaxel on tongue squamous cell carcinoma growth by inhibiting the JAK/STAT3 signaling pathway. Biomed Pharmacother. 2017;95:1388‐1396. 28946186 10.1016/j.biopha.2017.09.062

[9] Lu L , Zhang S , Li C , et al. Cryptotanshinone inhibits human glioma cell proliferation in vitro and in vivo through SHP‐2‐dependent inhibition of STAT3 activation. Cell Death Dis. 2017;8:e2767. 28492557 10.1038/cddis.2017.174 PMC5520699

[10] Pan Y , Shi J , Ni W , et al. Cryptotanshinone inhibition of mammalian target of rapa23in pathway is dependent on oestrogen receptor alpha in breast cancer. J Cell Mol Med. 2017;21:2129‐2139. 28272775 10.1111/jcmm.13135 PMC5571522

[11] Shen L , Zhang G , Lou Z , Xu G , Zhang G . Cryptotanshinone enhances the effect of Arsenic trioxide in treating liver cancer cell by inducing apoptosis through downregulating phosphorylated‐ STAT3 in vitro and in vivo. BMC Complement Altern Med. 2017;17:106. 28187727 10.1186/s12906-016-1548-4 PMC5303285

[12] Xu D , Lin TH , Li S , et al. Cryptotanshinone suppresses androgen receptor‐mediated growth in androgen dependent and castration resistant prostate cancer cells. Cancer Lett. 2012;316:11‐22. 22154085 10.1016/j.canlet.2011.10.006 PMC3283034

[13] Jiang G , Liu J , Ren B , et al. Anti‐tumor and chemosensitization effects of Cryptotanshinone extracted from Salvia miltiorrhiza Bge. on ovarian cancer cells in vitro. J Ethnopharmacol. 2017;205:33‐40. 28456578 10.1016/j.jep.2017.04.026

[14] Liu C , Sun HN , Luo YH , et al. Cryptotanshinone induces ROS‐mediated apoptosis in human gastric cancer cells. Oncotarget. 2017;8:115398‐115412. 29383168 10.18632/oncotarget.23267 PMC5777780

[15] Ke F , Wang Z , Song X , et al. Cryptotanshinone induces cell cycle arrest and apoptosis through the JAK2/STAT3 and PI3K/Akt/NFkappaB pathways in cholangiocarcinoma cells. Drug Des Devel Ther. 2017;11:1753‐1766. 10.2147/DDDT.S132488 PMC5479302 28670110

[16] Park IJ , Kim MJ , Park OJ , et al. Cryptotanshinone sensitizes DU145 prostate cancer cells to Fas(APO1/CD95)‐mediated apoptosis through Bcl‐2 and MAPK regulation. Cancer Lett. 2010;298:88‐98. 20638780 10.1016/j.canlet.2010.06.006

[17] Zhao J , Du P , Cui P , et al. LncRNA PVT1 promotes angiogenesis via activating the STAT3/VEGFA axis in gastric cancer. Oncogene. 2018 10.1038/s41388-018-0250-z 29706652

[18] Egusquiaguirre SP , Yeh JE , Walker SR , Liu S , Frank DA . The STAT3 Target Gene TNFRSF1A Modulates the NF‐kappaB Pathway in Breast Cancer Cells. Neoplasia. 2018;20:489‐498. 29621649 10.1016/j.neo.2018.03.004 PMC5916089

[19] Lin CH , Chiang MC , Chen YJ . STAT3 mediates resistance to anoikis and promotes invasiveness of nasopharyngeal cancer cells. Int J Mol Med. 2017;40:1549‐1556. 28949390 10.3892/ijmm.2017.3151

[20] Wang J , Xu J , Xing G . Lycorine inhibits the growth and metastasis of breast cancer through the blockage of STAT3 signaling pathway. Acta Biochim Biophys Sin (Shanghai). 2017;49:771‐779. 28910973 10.1093/abbs/gmx076

[21] Fei R , Zhang Y , Wang S , Xiang T , Chen W . alpha7 nicotinic acetylcholine receptor in tumor‐associated macrophages inhibits colorectal cancer metastasis through the JAK2/STAT3 signaling pathway. Oncol Rep. 2017;38:2619‐2628. 28901507 10.3892/or.2017.5935 PMC5780013

[22] Jia ZH , Jia Y , Guo FJ , Chen J , Zhang XW , Cui MH . Phosphorylation of STAT3 at Tyr705 regulates MMP‐9 production in epithelial ovarian cancer. PLoS ONE. 2017;12:e0183622. 28859117 10.1371/journal.pone.0183622 PMC5578655

[23] Herrera‐Perez RM , Voytik‐Harbin SL , Sarkaria JN , Pollok KE , Fishel ML , Rickus JL . Presence of stromal cells in a bioengineered tumor microenvironment alters glioblastoma migration and response to STAT3 inhibition. PLoS ONE. 2018;13:e0194183. 29566069 10.1371/journal.pone.0194183 PMC5863989

[24] Bournazou E , Bromberg J . Targeting the tumor microenvironment: JAK‐STAT3 signaling. JAKSTAT. 2013;2:e23828. 24058812 10.4161/jkst.23828 PMC3710325

[25] Chen Y , Fu LL , Wen X , et al. Sirtuin‐3 (SIRT3), a therapeutic target with oncogenic and tumor‐suppressive function in cancer. Cell Death Dis. 2014;5:e1047. 24503539 10.1038/cddis.2014.14 PMC3944233

[26] Ansari A , Rahman MS , Saha SK , Saikot FK , Deep A , Kim KH . Function of the SIRT3 mitochondrial deacetylase in cellular physiology, cancer, and neurodegenerative disease. Aging Cell. 2017;16:4‐16. 27686535 10.1111/acel.12538 PMC5242307

[27] Wang L , Wang WY , Cao LP . SIRT3 inhibits cell proliferation in human gastric cancer through down‐regulation of Notch‐1. Int J Clin Exp Med. 2015;8:5263‐5271. 26131100 PMC4483974

[28] Sun W , Liu C , Chen Q , Liu N , Yan Y , Liu B . SIRT3: a new regulator of cardiovascular diseases. Oxid Med Cell Longev. 2018;2018:7293861. 29643974 10.1155/2018/7293861 PMC5831850

[29] Meng G , Liu J , Liu S , et al. Hydrogen sulfide pretreatment improves mitochondrial function in myocardial hypertrophy via a SIRT3‐dependent manner. Br J Pharmacol. 2018;175:1126‐1145. 28503736 10.1111/bph.13861 PMC5866985

[30] Liu R , Fan M , Candas D , et al. CDK1‐Mediated SIRT3 Activation Enhances Mitochondrial Function and Tumor Radioresistance. Mol Cancer Ther. 2015;14:2090‐2102. 26141949 10.1158/1535-7163.MCT-15-0017 PMC4560959

[31] Li R , Quan Y , Xia W . SIRT3 inhibits prostate cancer metastasis through regulation of FOXO3A by suppressing Wnt/beta‐catenin pathway. Exp Cell Res. 2018;364:143‐151. 29421536 10.1016/j.yexcr.2018.01.036

[32] Xiong Y , Wang L , Wang S , et al. SIRT3 deacetylates and promotes degradation of P53 in PTEN‐defective non‐small cell lung cancer. J Cancer Res Clin Oncol. 2018;144:189‐198. 29103158 10.1007/s00432-017-2537-9 PMC11813316

[33] Cui Y , Qin L , Wu J , et al. SIRT3 Enhances Glycolysis and Proliferation in SIRT3‐Expressing Gastric Cancer Cells. PLoS ONE. 2015;10:e0129834. 26121691 10.1371/journal.pone.0129834 PMC4487898

[34] Wang Z , Liu Y , Lu L , et al. Fibrillin‐1, induced by Aurora‐A but inhibited by BRCA2, promotes ovarian cancer metastasis. Oncotarget. 2015;6:6670‐6683. 25749384 10.18632/oncotarget.3118 PMC4466642

[35] Schumacker PT . SIRT3 controls cancer metabolic reprogramming by regulating ROS and HIF. Cancer Cell. 2011;19:299‐300. 21397853 10.1016/j.ccr.2011.03.001 PMC3087169

[36] Li L , Chen CX . Thinking of neoadjuvant chemotherapy issues of advanced epithelial ovarian cancer. Zhonghua Fu Chan Ke Za Zhi. 2018;53:73‐76. 29534373 10.3760/cma.j.issn.0529-567X.2018.02.001

[37] Li M , Jin R , Wang W , et al. STAT3 regulates glycolysis via targeting hexokinase 2 in hepatocellular carcinoma cells. Oncotarget. 2017;8:24777‐24784. 28445971 10.18632/oncotarget.15801 PMC5421887

[38] Torrens‐Mas M , Pons DG , Sastre‐Serra J , Oliver J , Roca P . SIRT3 silencing sensitizes breast cancer cells to cytotoxic treatments through an increment in ROS production. J Cell Biochem. 2017;118:397‐406. 27420645 10.1002/jcb.25653

